# An interdomain helix in IRE1α mediates the conformational change required for the sensor's activation

**DOI:** 10.1016/j.jbc.2021.100781

**Published:** 2021-05-14

**Authors:** Daniela Ricci, Stephen Tutton, Ilaria Marrocco, Mingjie Ying, Daniel Blumenthal, Daniela Eletto, Jade Vargas, Sarah Boyle, Hossein Fazelinia, Lei Qian, Krishna Suresh, Deanne Taylor, James C. Paton, Adrienne W. Paton, Chih-Hang Anthony Tang, Chih-Chi Andrew Hu, Ravi Radhakrishnan, Tali Gidalevitz, Yair Argon

**Affiliations:** 1 Division of Cell Pathology, Children's Hospital of Philadelphia and University of Pennsylvania, Civic Center Boulevard, Philadelphia, Pennsylvania, USA; 2 Department of Biology, Drexel University, Philadelphia, Pennsylvania, USA; 3 Department of Biomedical and Health Informatics, Children's Hospital of Philadelphia, Philadelphia, Pennsylvania, USA; 4 Department of Bioengineering, University of Pennsylvania, Philadelphia, Pennsylvania, USA; 5 Research Centre for Infectious Diseases, Department of Molecular and Biomedical Science, University of Adelaide, Adelaide, South Australia, Australia; 6 Wistar Institute, Philadelphia, Pennsylvania, USA

**Keywords:** kinase RNase interdomain helix, RNase activity, IRE1 oligomerization, differential autophosphorylation, conformational change, BiP, binding protein, dox, doxycycline, ER, endoplasmic reticulum, HAP1KO, HAP1 cells deficient for IRE1α, IRE1α, inositol-requiring enzyme 1 α, MD, molecular dynamics, RIDD, regulated IRE1-dependent decay, Tm, tunicamycin, WT, wildtype

## Abstract

The unfolded protein response plays an evolutionarily conserved role in homeostasis, and its dysregulation often leads to human disease, including diabetes and cancer. IRE1α is a major transducer that conveys endoplasmic reticulum stress *via* biochemical signals, yet major gaps persist in our understanding of how the detection of stress is converted to one of several molecular outcomes. It is known that, upon sensing unfolded proteins *via* its endoplasmic reticulum luminal domain, IRE1α dimerizes and then oligomerizes (often visualized as clustering). Once assembled, the kinase domain trans-autophosphorylates a neighboring IRE1α, inducing a conformational change that activates the RNase effector domain. However, the full details of how the signal is transmitted are not known. Here, we describe a previously unrecognized role for helix αK, located between the kinase and RNase domains of IRE1α, in conveying this critical conformational change. Using constructs containing mutations within this interdomain helix, we show that distinct substitutions affect oligomerization, kinase activity, and the RNase activity of IRE1α differentially. Furthermore, using both biochemical and computational methods, we found that different residues at position 827 specify distinct conformations at distal sites of the protein, such as in the RNase domain. Of importance, an RNase-inactive mutant, L827P, can still dimerize with wildtype monomers, but this mutation inactivates the wildtype molecule and renders leukemic cells more susceptible to stress. We surmise that helix αK is a conduit for the activation of IRE1α in response to stress.

The endoplasmic reticulum (ER) is highly specialized for the folding and quality control of secreted, plasma membrane, and organelle proteins. Accumulation of proteins in the ER initiates a cellular response called unfolded protein response, a signal transduction and transcriptional program that increases the folding capacity of the cell in an attempt to alleviate the stress. If the stress is not resolved, the cell is committed to apoptosis (1). ER stress can be induced chemically (*e.g.*, by inhibiting the ER calcium pump with thapsigargin, by inhibiting glycosylation with tunicamycin [Tm], or by blocking disulfide bond formation with dithiothreitol) or can be induced by different physiological processes. Professional secretory cells, such as pancreatic β cells and plasma cells, which produce high levels of proteins, are particularly prone to physiological ER stress (2, 3, 4, 5, 6).

Prompt sensing of ER stress is important to enable the proper coping response. In mammalian cells, ER stress is detected by three different ER transmembrane sensors: inositol-requiring enzyme 1 α (IRE1α), protein kinase RNA–like ER kinase, and activating transcription factor-6. The IRE1α arm is the most conserved one and has been extensively studied. Two mechanisms have been proposed for its activation by ER stress: direct binding to misfolded proteins (7) or the dissociation of the ER luminal chaperone, the binding protein (BiP/GRP78), which keeps IRE1α inactive (8, 9) and sequesters it in this form (10). IRE1α then dimerizes through its luminal domain (11) and undergoes autotransphosphorylation *via* its cytoplasmic kinase domain (12). This in turn triggers a conformational change that activates the RNase domains of IRE1α (13). The enzymatically active IRE1α either cleaves XBP1 mRNA at two sites, to create the reading frame for the active XBP1 transcription factor, or cleaves a select group of transcripts once each, initiating regulated IRE1-dependent decay (RIDD) of these transcripts (14).

The sequence of events that activates IRE1α was defined by showing that mutations in the luminal domain, in the cytosolic kinase domain or in the cytosolic RNase domain can render IRE1α inactive and abrogate the downstream signaling. How the three domains of the molecule form a modular activation relay is not yet understood. Deciphering this mechanism is important because IRE1α can be activated not only by luminal ER stress but also by membrane perturbations that do not require the luminal domain (15), as well as by a bypass mode in response to binding of flavonoids to the cytosolic dimer interface of the RNase domains (16).

The critical event during mammalian IRE1α activation was long thought to be the phosphoryl transfer reaction (17). Counterintuitively, however, some kinase-inactive IRE1α mutants can support RNase activity if provided with appropriate nucleotide mimetics that bind in the kinase pocket, but are not hydrolyzed (18, 19, 20). This indicates that the key event is not the phosphoryl transfer *per se* but the conformational change that ensues. The nature of this conformational change, however, is currently not defined. Yeast IRE1α had been crystalized in two states: an RNase-inactive conformation with the kinase sites of the two monomers oriented “face to face” and an RNase “active” back-to-back conformation (17, 21, 22). Whether a similar rotation of the entire luminal portion of mammalian IRE1α is the relevant conformational change that governs its activation or whether there are other changes that convey the active state of the kinase domain to the RNase domain is currently unknown. Therefore, it is important to define the intramolecular changes within IRE1α that occur after the kinase activation and initiate RNase activity.

Toward this end, we characterized mutations in helix αK, connecting the kinase and RNase domains, which unexpectedly affected the enzymatic activities of IRE1α despite being far removed from either of the two active sites. We show here that some substitutions in helix αK, specifically of residue L827, render IRE1α enzymatically inactive, without inhibiting other activities such as dimerization and also inhibits a wildtype version of IRE1α. Of importance, mutations in helix αK have long-range effects on the conformation of IRE1α when it is activated by ER stress, suggesting that the interface between the kinase and RNase domain of IRE1α might tune distinct outcomes of IRE1α activities.

## Experimental procedures

### Cell culture and reagents

A derivative of HAP1, a near-haploid human cell line, termed HAP1KO, was engineered by CRISPR-Cas9 editing, to abolish the expression of IRE1α. HAP1 and HAP1KO cells were maintained in IMDM medium (Invitrogen) (see (23) for more details). Kms11 is a human myeloma line (24) that was grown in RPMI 1640 (Mediatech) medium with 10% fetal bovine serum, 1% penicillin/streptomycin, and 1% sodium pyruvate (Sigma).

HAP1, HAP1KO, and Kms11 cells were transduced with a Tet-On lentivirus and with a pTIGHT-IRE1-GFP-HA (IRE1GFP) lentivirus and selected for antibiotic resistance as in (23). Where indicated, IRE1GFP WT and mutants with only GFP or HA tag were also used. IRE1GFP expression was induced with 1 μg/ml doxycycline (dox) (Biochemika) overnight.

Tunicamycin and 4μ8c were from Calbiochem, thapsigargin was from MP Biomedicals, and Luteolin was from Sigma. GFP-Trap A beads were from Chromotek, and trypsin was from Promega.

### Mutagenesis

The pTIGHT-IRE1-GFP-HA WT plasmid was used as a template for site-directed mutagenesis according to (25). Pfu Ultra II Fusion HS polymerase was purchased from Agilent. All mutations were validated by Sanger sequencing. Primers used: L827P: TCAGCGAAGCACGTGGCCAAACACCCGTTCTTC; P827L: GAAGCACGTGCTCAAACACCCGTTCTT; L827F: TCAGCGAAGCACGTGTTCAAACACCCGTTCTTC; L827Q: TCAGCGAAGCACGTGCAGAAACACCCGTTCTTCTG; L827R: TCAGCGAAGCACGTGCGCAAACACCCGTTCTTC; D123P: CTCTACATGGGTAAAAAGCAGCCCATCT.

### *In silico* analysis of protein structure

Rosetta (release 3.11) was used to predict the changes in protein stability due to the point mutations. The input was the crystal structure of apo human IRE1α (Protein Data Bank [PDB]: 5HGI). It was preminimized using the “minimize_with_cst” application in Rosetta. We followed a ΔΔG _monomer application described by Kellogg *et al*. (26) for estimating stability changes in monomeric proteins in response to point mutations. In brief, this application uses the input structure of the WT protein to generate a structural model of the point mutant. The ΔΔG is given by the difference in Rosetta energy unit between the WT structure and the point mutant structure. For more precise calculation, 50 models for each WT and mutant structures were generated, and the most accurate ΔΔG was calculated as the difference between the mean of the top three scoring WT structures and the top three scoring point-mutant structures. The “show_bumps” plugin in PyMol was used to depict the potential steric hinderance and van der Waals clashes in the WT and modeled structures.

### RNA extraction, PCR, and qPCR

Total RNA was isolated with Trizol (Life Technologies), following manufacturer's instructions. RNA, 200 ng, was retrotranscribed to cDNA by priming with oligo(dT)12-18 and Superscript II retrotranscriptase (Invitrogen). Primers to detect human unspliced/spliced XBP1: fwd: AAACAGAGTAGCAGCTCAGACTGC; rev: TCCTTCTGGGTAGACCTCTGGGAG. Quantitative PCR was performed using SYBR green reagent (Applied Biosystems) and the reaction run on Applied Biosystems StepOne Plus machine. Data were analyzed using the delta-delta-Ct method. qPCR primers: Rpl19: fwd: AAAACAAGCGGATTCTCATGGA; rev: TGCGTGCTTCCTTGGTCTTAG; Blos1: fwd: CAAGGAGCTGCAGGAGAAGA; rev: GCCTGGTTGAAGTTCTCCAC; CHOP: fwd: GGAGCTGGAAGCCTGGTATG; rev: AAGCAGGGTCAAGAGTGGTG.

### Immunoprecipitation

Cells were lysed in lysis buffer (50 mM Tris-HCl pH 8, 150 mM NaCl, 5 mM KCl, 5 mM MgCl2, 1% NP-40, 20 mM iodoacetamide, 1X protease inhibitors [Roche]). Five percent of the volume of the lysate was saved as “input” in sample buffer, and the rest was diluted in TNNB buffer (50 mM Tris pH 7.5, 250 mM NaCl, 0.5% NP-40, 0.1% BSA, 0.02% NaN_3_). GFP-Trap_A beads were added and incubated for 1 h at 4 °C. After washing, beads were resuspended in sample buffer and boiled for 5 min, and the proteins were analyzed by SDS-PAGE and Western blot.

### Western blots

Cells were lysed in lysis buffer (50 mM Tris-HCl pH 8, 150 mM NaCl, 5 mM KCl, 5 mM MgCl_2_, 1% NP-40, 20 mM iodoacetamide, 1X protease inhibitors [Roche]). The protein content was determined by BCA assay (Pierce), and proteins were separated by SDS-PAGE and transferred onto nitrocellulose membrane (Bio-Rad). Membranes were blocked, probed with primary and secondary antibodies, and scanned on an Odyssey Infrared imager (Li-Cor).

Primary antibodies used: anti-IRE1α (Cell Signaling Technology); anti-HA (Covance); anti-14.3.3 (Santa Cruz); anti-phospho-IRE1α Ser724 (Novus Biologicals); anti-phospho-IRE1α Ser729 (Dr Hu, Wistar Institute, Philadelphia, PA). IRDye-conjugated secondary antibodies were from Li-Cor.

### Limited proteolysis

Cells were lysed in lysis buffer (50 mM Tris-HCl pH 8, 150 mM NaCl, 5 mM KCl, 5 mM MgCl_2_, 1% NP-40, 20 mM iodoacetamide), the protein content was determined as above, and trypsin was added at the indicated final concentration and incubated on ice for 30 min. The reaction was stopped by adding sample buffer and boiling the samples for 5 min. Western blot was then performed as above.

### Microscopy and image analysis

HAP1 cells were plated on 35-mm microscopy-grade plastic dishes (Ibidi). After dox induction and subsequent imposition of chemical ER stress, the cells were imaged over 8 h using a Marianas fluorescence microscope equipped with an OKO Lab CO_2_ enclosure on a Zeiss inverted platform, with a 63X Plan-Apochromat 1.4 NA objective. Images were collected as in (23). Exposure times varied between 0.1 and 0.5 s, depending on sample intensity, unless otherwise specified. In some experiments, cells were imaged using a 63X Plan Apo 1.4 NA objective on an Axiovert 200M (Zeiss) with a spinning disc confocal system (UltraVIEW ERS6, PerkinElmer). Images were collected using an ORCA Flash 4.0 camera (Hamamatsu Photonics) using Volocity V.6.3.1 acquisition software.

### Colony formation assay

HAP1 or HAP1 L827P IRE1GFP cells were plated in six-well plates (Corning) at 5000 cells per well. IRE1GFP expression was induced with dox, and medium was changed every 2 days with fresh dox. Cells were stressed with various concentrations of Tm for the indicated times. The drug was then washed out, and the plates were incubated for 6 days at 37 °C and were then stained with 0.2% (w/v) crystal violet (Sigma) in 2% ethanol for 1 h at room temperature. The crystals were then dissolved in 2% SDS (1 h), and the color was quantified at OD570.

### Statistical analyses

To enumerate cells containing clusters, >200 cells were counted per condition in two or more independent experiments. Images were analyzed and quantified using a homemade cluster analyzer for ImageJ (23). Statistically significant differences between normally distributed populations were evaluated by Student's *t* test and by nonparametric tests, when the distributions were nonnormal.

### Molecular dynamics simulations

Molecular dynamics (MD) simulations were performed on PDB files of mammalian IRE1α using the GROMACS 2018.8 software, with the AMBER14SB force field. The initial monomeric model was based on the crystal structure of human kinase and RNase domains in complex with ADP (PDB: 3P23, chain A). The User Template Mode of Swiss-Model web was employed to rebuild the missing structures in the model. After the removal of ADP and Mg^2+^ in the rebuilt monomeric structure, UCSF Chimera was used to align the rebuilt monomer structure to chains A and B in original 3p23.pdb and to build the complete face-to-face IRE1α dimer.

MD simulations followed previously described methods (27). The 3p23 dimer was solvated in a cubic periodic box with TIP3P waters, and Na^+^ and Cl^−^ were added to 0.1 M to neutralize the system. The energy minimization step was applied to the system until maximum force was less than 1000 kJ/mol/nm. Then, 100 ps Number of particles, volume, and temperature equilibration and 100 ps normal pressure and temperature equilibration were used to equilibrate the system. A 400-ns simulation was performed under normal pressure and temperature condition, using the sd integrator with 2-fs time step. The simulation temperature was set to 300 K for all simulations. The dimers of the L827P and L827F mutants were built using the Structure Editing function in UCSF Chimera. Each simulation of WT, L827P, and L827F was done in duplicate.

## Results

### The interdomain mutation L827P renders IRE1α inactive

To address how the activation signal is transmitted from the kinase domain of IRE1α to its RNase domain, we focused on a mutation that was discovered in a random mutant screen, L827P, that drastically inactivated this stress sensor despite being far removed from either the kinase or the RNase active sites. L827P is located in the vicinity of another previously reported mutation, P830L, which also renders IRE1α inactive (28). The location of both mutations in between the two functional domains suggested that this area might be important for IRE1α activity and perhaps for transmitting a signal from the kinase to the RNase domains.

**Figure 1:**
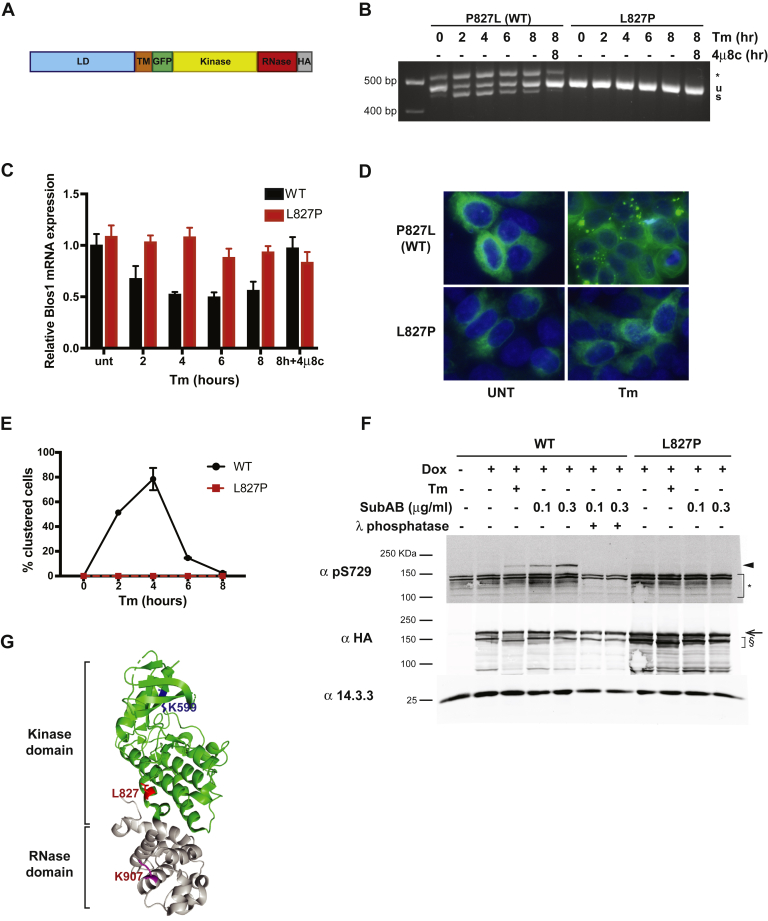
**IRE1α L827P is an enzymatically inactive mutant**. *A*, structure of the GFP and HA-tagged human IRE1α used in this work. The construct contains the luminal domain (LD) and transmembrane domain (TM). The superfolded GFP (GFP) is grafted in-frame between codons 494 and 495 in the cytosolic portion of the molecule. Following the kinase and RNase domains, three HA peptides are inserted at the C terminus of IRE1α. *B*, IRE1α L827P fails to support the XBP1 splicing activity. HAP1KO cells were complemented by either the revertant WT human IRE1GFP (P827L) or the L827P mutant. Each stable subline was subjected to treatment with 4 μg/ml Tm for the indicated times, after which splicing of XBP1 transcripts was assayed by an RT-PCR gel assay. Specificity of the assay for IRE1α activity was assessed by inclusion of 4μ8c (16 μM) where indicated. ∗, hybrid band resulting from unspliced and spliced XBP1 (46). *C*, IRE1α L827P fails to support regulated IRE1-dependent decay activity. The same stable sublines were stressed as in *B*, and then the regulated IRE1-dependent decay activity of IRE1α was assayed using qPCR quantitation of the common BLOC1S1 substrate relative to the unaffected ribosomal gene Rpl19. *D*, IRE1GFP L827P does not cluster in response to endoplasmic reticulum stress. HAP1KO IRE1GFP WT or L827P cells were treated with Tm (4 μg/ml) and imaged over 8 h. Images are representative fields of the 4-h treatment. *E*, quantification of clustering. The images from *D* were quantified and plotted. *F*, L827P IRE1GFP is not phosphorylated on S729 following induction of endoplasmic reticulum stress. HAP1KO IRE1GFP WT or L827P was stressed with Tm as in *D* and with SubAB at the indicated concentrations for 2 h. Cells were lysed and activation of IRE1α was assessed by Western blot with an antibody against phosphorylated Ser729 of IRE1α. 14.3.3, housekeeping protein. *Arrow*, full-length IRE1GFP; *arrowhead*, phospho-IRE1GFP S729; §, lower-molecular-weight bands that appear to be IRE1α specific and size-sensitive to Tm treatment; ∗, nonspecific bands. G. Location of L827 in the crystal structure of IRE1α. Residue L827 of human IRE1α is highlighted in *red* in Protein Data Bank 5HGI. For orientation, the catalytic residue in the kinase domain, K599, is marked in *blue* and the catalytic residue in the RNase domain, K907, is marked in *purple*. U, unspliced XBP1; s, spliced XBP1.

In order to study the mutant's effect on IRE1α activity, we used a clone of HAP1 cells deficient for IRE1α (HAP1KO) as a host for functional complementation of IRE1α and analysis of structure–function relationships (23). HAP1KO cells only activate the XBP1 transcription factor when transduced with active IRE1α (23), such as Dox-inducible GFP- and HA-tagged IRE1α (Fig. 1, *A* and *B* and (23)).

Expression of IRE1α L827P failed to support XBP1 splicing in HAP1KO cells (Fig. 1*B*). When P827 was mutated back to the wildtype (WT) Leu, stress-dependent splicing activity was restored (Fig. 1*B*, denoted P827L), showing that the inactivation of IRE1α was due to the L827P substitution. In addition to its inability to support XBP1 splicing, L827P also failed to perform the RIDD activity of IRE1α, as measured by the transcript levels of the known target BLOC1S1 (Fig. 1*C*). Thus, the mutant IRE1α L827P is unable to perform either of the RNase activities. Furthermore, IRE1α L827P failed to cluster in response to ER stress, even at late time points (Fig. 1, *D* and *E*).

All the activities that are defective in the L827P mutant should depend on the autophosphorylation activity of IRE1α (23, 29, 30). Therefore, we tested the phosphorylation status of L827P IRE1α using an antibody that detects phospho-Ser729 (30). Treatment of cells with Tm induced phosphorylation of WT IRE1α, and an even more robust phosphorylation was evident upon ablation of BiP with the toxin SubAB, a treatment known to provide higher level of ER stress (Fig. 1*F*; (31)). In contrast, no significant phospho-Ser729 was detected for L827P IRE1α even at a high concentration of SubAB (Fig. 1*F*). Because of the specificity of the antibody, it is possible that L827P IRE1α is phosphorylated elsewhere but not on Ser729. Therefore, we subjected ER-stressed cell lysates to lambda phosphatase and resolved them by electrophoresis to visualize the migration shifts. This gel shift assay, in which proteins (treated or not with lambda phosphatase) are resolved on a long gel, is comparable with Phos-Tag gels in dissecting IRE1α phosphorylation. As shown in Figure S1*A*, WT IRE1α resolves into two species, with the higher-molecular-weight one being phosphorylated. Tm-treated or SubAB-treated IRE1α L827P exhibited no significant mobility shifts compared with unstressed IRE1α, indicating that the L827P protein is indeed not phosphorylated upon ER stress.

The P830L mutation was previously shown to render IRE1α less stable, and that was proposed as a possible explanation for its drastic phenotype (28). However, L827P showed a similar half-life to WT IRE1α (Fig. S1*B*), suggesting that unlike P830L, L827 affects the activity of IRE1α but not its stability.

We conclude that L827P specifies a form of IRE1α that lacks most of the activities of the protein, even though the mutation is spatially distant from the IRE1α catalytically active sites (Fig. 1*G*).

### L827P IRE1α is a dominant negative mutant

**Figure 2:**
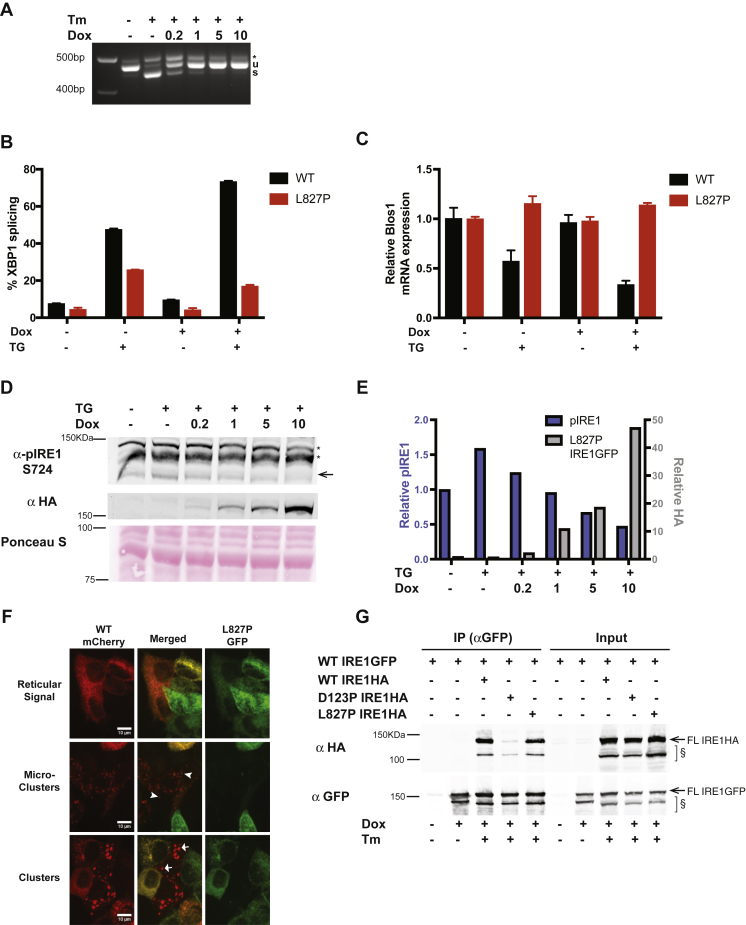
**The L827P mutant inhibits WT IRE1α in HAP1 and Kms11 cells**. *A*, IRE1GFP L827P inhibits XBP1 splicing in response to Tm in leukemic HAP1 cells. Parental HAP1 cells expressing IRE1GFP L827P in addition to the endogenous WT IRE1α were induced with the indicated concentrations of dox (μg/ml). The cells were then treated with 4 μg/ml Tm for 4 h, and XBP1 splicing was assessed by RT-PCR. *B*, L827P IRE1GFP inhibits ER stress-induced XBP1 splicing in multiple myeloma Kms11 cells. Kms11 cells expressing WT or L827P IRE1GFP were treated with 0.5 μM Tg for 4 h where indicated. RNA was extracted and XBP1 splicing was assessed by RT-PCR and quantified. *C*, L827P inhibits regulated IRE1-dependent decay activity in response to ER stress in Kms11 cells. The same samples as in *B* were used to perform a qPCR to detect BLOC1S1 expression levels as in Figure 1*C*. *D*, expression of L827P decreases endogenous WT IRE1α phosphorylation in response to ER stress. Parental HAP1 cells, with constitutive expression of WT IRE1α and inducible expression of IRE1GFP L827P were induced with dox and treated with Tg (0.2 μM for 4 h). Cells were lysed and proteins were analyzed by Western blot. *Arrow*, endogenous phospho-S724 IRE1α; ∗, nonspecific bands. E. Quantification of phospho-IRE1α S724 and L827 mutant. The intensities of the Western blot bands from the experiment described in *D* were normalized to total protein contents of the samples, measured by Ponceau S staining, quantified, and plotted. *F*, L827P inhibits WT IRE1α clustering. Representative images of WT IRE1mCherry/L827P IRE1GFP-expressing cells treated with 4 μg/ml Tm for 3 h. Cells that coexpress mCherry and GFP typically exhibit reticular signal with no clusters. mCherry-expressing cells with low GFP expression form faint cluster-like foci termed dim clusters or the bright foci typical of WT IRE1α clusters. *Arrowheads*, two typical dim clusters; *arrows*, two typical bright clusters. *G*, L827P binds full-length WT IRE1α. HAP1KO cells were recomplemented with WT IRE1GFP containing only the GFP tag, WT IRE1HA, D123P IRE1HA, L827P IRE1HA containing only the HA tag or combinations of constructs. The cells were induced with dox and treated with 4 μg/ml Tm for 4 h where indicated. Cells were collected, lysed, and subjected to immunoprecipitation with GFP-Trap beads. Beads-bound proteins were analyzed by Western blot. Input: 5% of the lysates. *Arrow*, full-length IRE1GFP or IRE1HA; §, lower-molecular-weight bands that appear to be IRE1α specific and size sensitive to Tm treatment. ER, endoplasmic reticulum.

Since the L827P mutant is enzymatically inactive, we next tested whether L827P could interact and influence the activity of WT IRE1α. We expressed dox-inducible L827P IRE1GFP in the parental HAP1 cell line that had intact endogenous IRE1α. Prior to induction of the mutant protein with dox, these cells exhibited canonical XBP1 splicing activity when treated with Tm (Fig. 2*A*). However, when L827P IRE1GFP expression was induced, the XBP1 splicing activity of the WT allele was progressively inhibited in proportion to the level of induction of L827P IRE1GFP (Fig. 2, *A* and *D*).

To test if this inhibitory effect was cell-type specific, we expressed WT or L827P IRE1GFP in multiple myeloma Kms11 cells that express intact endogenous IRE1α, do not have high basal activation of IRE1α, and are responsive to ER stress. As shown in Figure S2, some exogenous IRE1GFP was expressed at a basal level in the absence of Dox, and its level then increased upon addition of dox. For this reason, both before and after ER stress was induced with Tg for 4 h, L827P IRE1GFP inhibited activity of the endogenous IRE1α, with inhibition being the strongest when its expression was increased by dox treatment, showing only ∼16% XBP1 splicing (Fig. 2*B*). A similar inhibition was observed for the RIDD activity: in the presence of L827P protein, endogenous IRE1α was unable to cleave Blos1 in response to Tg treatment, whereas in the presence of WT IRE1GFP, Blos1 mRNA was reduced by 60% (Fig. 2*C*) in multiple myeloma cells.

Furthermore, coexpression of L827P IRE1GFP, which does not autophosphorylate (Figs. 1*F* and S1*A*), inhibited the phosphorylation activity of the endogenous WT IRE1α (assayed by phosphorylation of serine 724) in a dose-dependent manner (Fig. 2, *D* and *E*). Dox dose-dependent inhibition of the endogenous IRE1α reflected increasing levels of the mutant protein (Fig. 2, *D* and *E*). Thus, the mutant IRE1α inactivates the endogenous WT IRE1α, showing a dominant-negative effect. This inhibition is likely by direct interaction with WT IRE1α, rather than by affecting ER stress signaling, as shown by insensitivity of CHOP transcription to the expression of L827P (Fig. S3).

Clustering of WT IRE1GFP is a function that is distinct from the RNase enzymatic activity (23); since the dominant-negative effect of the mutant protein suggested an interaction with the WT protein, we asked whether L827P IRE1GFP affects clustering of WT IRE1α. Upon Tm stress, most cells coexpressing WT IRE1mCherry with L827P IRE1GFP failed to exhibit clusters (Fig. 2*F*), indicating that the presence of L827P IRE1α in the same cell inhibits clustering of WT molecules. Therefore, L827P IRE1GFP is dominant negative not only with respect to kinase and RNase activities but also with respect to stress-induced oligomerization of IRE1α.

To confirm whether the dominant-negative mechanism is mediated by direct interaction, we created two different versions of exogenous IRE1α: tagged with GFP only (IRE1GFP) or with HA only (IRE1HA). We then coexpressed L827P IRE1HA and assessed its association with WT IRE1GFP, *via* immune isolation with anti-GFP beads. As shown in Figure 2*G*, association of L827P IRE1α with WT IRE1α happens to the same extent as association of two WT monomers. By comparison, the dimerization-impaired D123P IRE1α mutant (32) associated with WT IRE1α much less efficiently. Thus, the L827P mutant protein inhibits the WT IRE1α by forming mixed dimers.

### The L827P mutation reduces the ability of HAP1 cells to cope with ER stress

**Figure 3:**
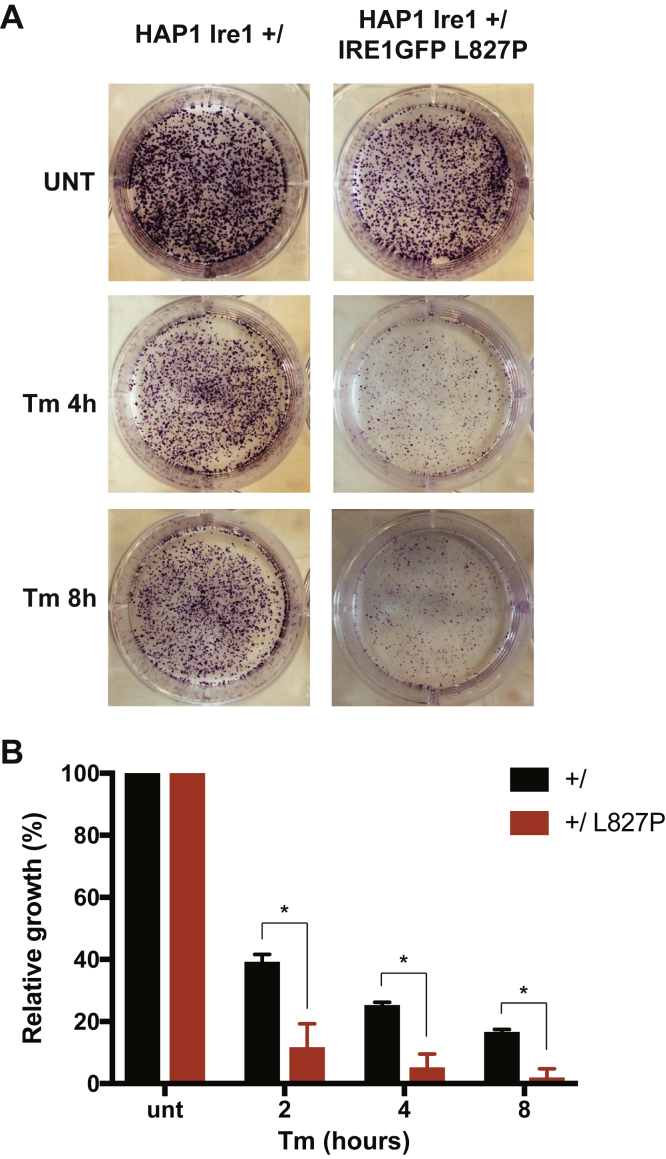
**The L827P mutant renders cells expressing endogenous IRE1α more sensitive to endoplasmic reticulum stress**. *A*, the L827P mutant increases the sensitivity of leukemic HAP1 cells to endoplasmic reticulum stress. Parental HAP1 cells expressing endogenous, WT IRE1α and HAP1 cells expressing both endogenous WT IRE1α and L827P IRE1GFP were induced with dox and then treated with 0.5 μg/ml Tm for the indicated time, after which the cells were washed and allowed to grow for 6 days. Colonies that developed by that time were stained with crystal violet. B. Quantification of cell growth. The crystals from the plates shown in A were dissolved in 2% SDS and the *A*_570_ of the solutions were quantified. ∗*p* < 0.05.

Chronic activation of IRE1α is known to be proapoptotic (33), and many enzymatically inactive IRE1α mutants are deficient for apoptosis, whereas others are even able to rescue cells from ER stress-mediated apoptosis by inhibiting the activation of the endogenous WT protein (20, 34). Of interest, although the P830L mutation near residue L827 causes loss of the enzymatic activities, it has only subtle inhibitory effect on XBP1 splicing by the WT IRE1α (28) when overexpressed and does not slow cell growth (20). Therefore, we asked whether the dominant-negative inhibition by L827P has important consequences for ER stress resistance. To avoid the confounding effects of chronic stress, we used a colony formation assay, where cells were exposed to a brief period of ER stress, washed, replated in normal growth medium, and then evaluated for growth 6 days later. As shown in Figure 3*A*, a mild dose of Tm (0.5 μg/ml for 4 h) was sufficient to inhibit HAP1 colony growth. Expression of the L827P IRE1GFP protein in these cells caused a further dramatic decrease in cell survival in response to the Tm stress (Fig. 3*B*). This was unexpected, given that other enzymatically inactive mutants either do not affect cell survival or protect cells from apoptosis (20, 34).

We conclude that unlike previously reported inactivating mutations, L827P expression reduces the cells' ability to cope with ER stress, likely owing to its interaction with WT IRE1α in *trans*.

### Different substitutions at position 827 affect IRE1α activities differentially

**Figure 4:**
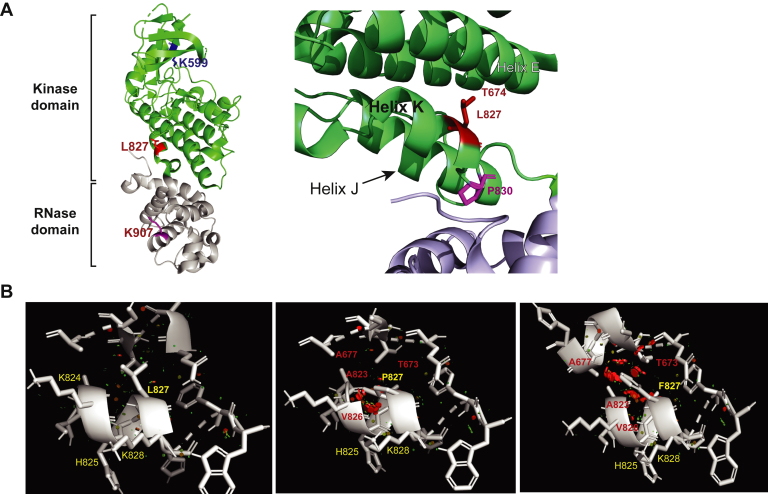
**Structural elements of the interdomain helix αΚ**. *A*, crystal structure of the cytoplasmic portion of human IRE1α (from PDB 5HGI). Kinase domain residues are colored *green* and RNase domain residues are colored *light blue*. *Left*, overall structure with residue L827 (in *red*), as well as the catalytic kinase residue K599 and the RNA catalytic residue K907 depicted in stick format and highlighted in *blue* or *purple*, respectively. *Right*, close-up view of the interdomain helix and L827, which projects from helix αK toward T674 in helix αE. P830 is proximal to the RNase domain. *B*, predicted intramolecular clashes when Leu827 is mutated to either Pro827 or Phe827. WT IRE1α (Leu827, *left*) was substituted *in silico* with Pro827 (*center*) or Phe827 (*right*), and the amino acids within 5 Å are depicted. In each case, neighboring residues with predicted molecular clashes (Rosetta) are shown in *red*, whereas neighbors without predicted clashes are colored *yellow*.

We next asked why the IRE1α L827P mutation had such a dramatic effect on IRE1α activities and cellular phenotype. The L827P mutation is located near the previously identified mutant P830L, at the interface between the kinase and RNase domains at the end of helix αk (S821 to P830 in PDB 4PL3) (Fig. 4*A*). Because of the proximity of the two mutations, we hypothesized that the integrity of helix αK is necessary for thermodynamic stability and for attaining the active conformation of the IRE1α RNase domain.

Residue L827 is relatively solvent exposed and is packed against Thr674 from helix αE (Fig. 4*A*), in a relatively hydrophobic surface (residues 665–680 of murine IRE1α (35); residues 664–681 of human IRE1α [PDB 5HGI; (36)]). The conformation of Leu827 and the interactions between helices αK, αE, and αJ are similar in crystals of the murine or human IRE1α (Fig. 4*A*) and, importantly, are not changed by binding of nucleotides or inhibitors to the kinase domain, nor by binding of flavonoids to the RNase domain (16). Therefore, we postulated that there are conformational changes that are needed to attain the active IRE1α RNase domain but are not captured in the current protein crystals.

To address this possibility, we modeled the cytosolic domains of IRE1α using the algorithms Rosetta (26) and SDM (37) that predict the changes in protein thermodynamic stability upon substitutions of single amino acids. The Rosetta-predicted structural changes, based on the 5HGI crystal structure of human IRE1α, showed that Pro is the most destabilizing substitution at position 827, with an estimated ΔΔG at −2.35 units (Table 1). The substitution of Pro for Leu827 is very likely to cause perturbation in the local conformation of neighboring amino acids, as mapping Pro827 on the structure of human (PDB 5HGI) or murine (PDB 4PL3 or 4PL5) IRE1α suggests that this substitution would clash with Val826, Lys824, and Ala823, the residues that form the turn connecting helices αJ and αK (Fig. 4*B*). This would weaken the interaction of helices αK-αE and change the geometry of helix αK, at least with respect to Val826, His825, and Lys824. It is quite possible that these main-chain alterations could propagate to the RNase active site (Fig. 4*A* and see more below).

**Table 1:** Predicted thermodynamic stability of IRE1α mutants

Mutation	Rosetta (energy units)	SDM ΔΔG
L827P	ND	−2.23
L827P	234.6	−2.75
L827I	3.0	−0.45
L827V	4.7	−2.93
L827F	109.1	−1.14
L827Y	6.9	−0.93
L827R	6.3	−1.35
L827Q	6.1	−0.29
L827E	7.7	−0.21
L827A	5.7	−2.69
P830L	40.0	ND
P830A	3.8	ND
K907A	ND	−0.56
K599A	ND	−0.38

Substitutions of Leu827 characterized in this work and the P830 mutations characterized in (28) were modeled in the 5HGI PBD structures of human IRE1α, and the predicted free energy changes calculated according to the Rosetta algorithm (26). For comparison, many mutations were also modelled in the 4PL5 PDB structure of murine IRE1α and their predicted free energy changes were calculated according to the SDM algorithm (37). ND, not determined. The destabilization energies of the RNase active site (K907A) and the kinase active site (K599A) also serve as useful comparisons.

The Rosetta algorithm also predicts that, Phe827, in addition to Pro827, is a destabilizing substitution. All other substitutions are far less destabilizing (Table 1). Modeling Phe827 suggests that three-fourths of its common rotamers would clash with Thr673 in helix αE or with Asp824 and Val826 in helix αK. The Phe rotamer with the fewest clashes is shown in Figure 4*B*. Therefore, modeling predicted that different substitutions of Leu827 would have a graded loss-of-function phenotype.

**Figure 5:**
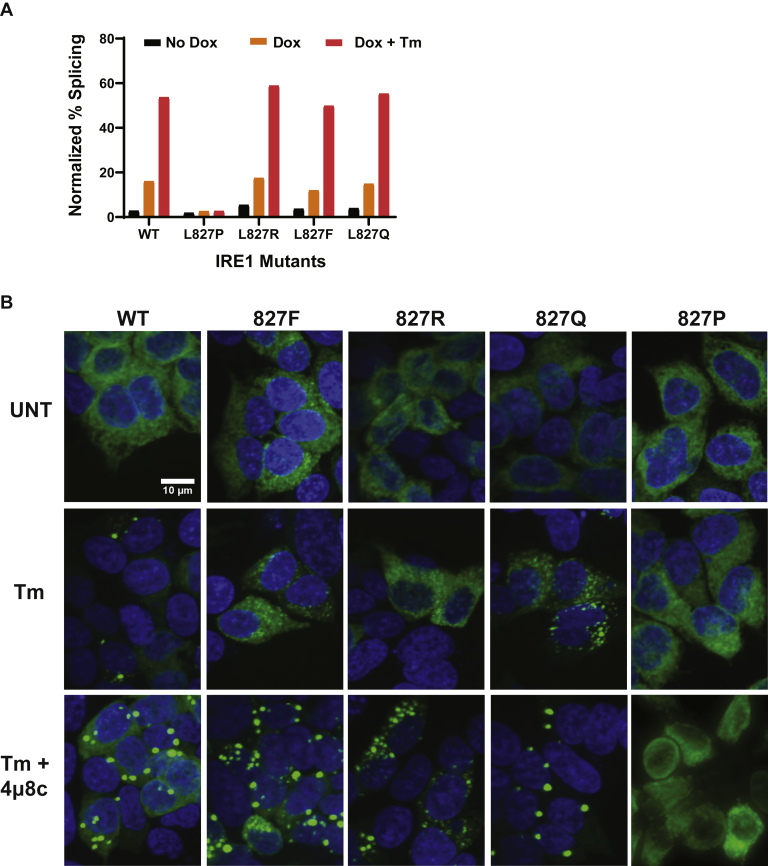
**Graded RNase activity and clustering in response to substitutions of L827**. A. RNase activity of L827 substitutions. HAP1KO IRE1GFP WT or L827 mutants were treated with 4 μg/ml Tm for 4 h to activate XBP1 splicing where indicated. The percentage of splicing was measured from RT-PCR analysis and normalized to the protein expression as measured in Figure S4. *B*, clustering behavior of L827 substitutions. Representative fields of stable clones that were subjected to ER stress (Tm, 4 μg/ml) for 4 h, or left untreated (UNT) and imaged. A combined treatment of Tm plus 4μ8c (16 μM) was used to generate the mega clusters, as described by Ricci *et al*. (23).

To test some of these predictions, we generated additional IRE1α mutants with substitutions with Arg, Gln, and Phe at residue 827. The protein expression levels of the mutants were comparable with WT IRE1α (Fig. S4). When assayed in HAP1KO cells, L827P was the most severe substitution, displaying no detectable XBP1 splicing activity (Figs. 1 and 5*A*). On the other hand, L827R, L827F, and L827Q IRE1α had similar XBP1 splicing activities and were indistinguishable from the WT protein (Fig. 5*A*).

A second activity we tested was clustering of IRE1α. As we showed earlier, L827P is clustering deficient, whereas under the same conditions of Tm stress, WT IRE1α forms bright foci (Figs. 1 and 5*B*). The different Leu827 substitutions display distinct clustering behaviors; L827R and L827F IRE1α, despite being fully active, are not able to form bright foci in response to Tm treatment (Fig. 5*B*). The L827Q did form clusters that were similar in size and number per cell to the WT clusters, but they were duller than clusters of WT IRE1α, and a smaller fraction of the cellular IRE1α was incorporated into them (more IRE1α remained reticular) (Figs. 5*B* and S5), suggesting that, although L827Q molecules are active, they do not pack as efficiently into clusters as WT. These data suggest that sequence variation at position 827 has divergent effects on the protein's ability to respond to ER stress by dimerization, oligomerization, and activation of RNase activity.

The decreased clustering of the F, R, and Q substitutions posed a question whether these proteins were not able to access a conformation that is needed for oligomerization, or whether they formed labile oligomers. As we showed previously (23), enzymatically inactive IRE1α still forms clusters, and, in fact, the IRE1α RNase inhibitor 4μ8c causes formation of larger and more persistent clusters. Therefore, we asked whether these mutants could cluster when inhibited. Indeed, L827R, L827F, and L827Q responded to 4μ8c+Tm by forming foci that were indistinguishable from the large clusters formed by similarly treated WT IRE1α (Fig. 5*B*). Thus, the substitutions L827R, L827F, and L827Q were inherently competent to cluster but defective in their response to Tm stress. Of interest, the L827P protein did not cluster when inhibited, suggesting that this substitution induces a conformation that is capable of dimerizing, based on its dominant-negative behavior, but not oligomerizing.

### Clustering-impaired L827F does not affect IRE1α RNase activities but has a distinct phosphorylation pattern

**Figure 6:**
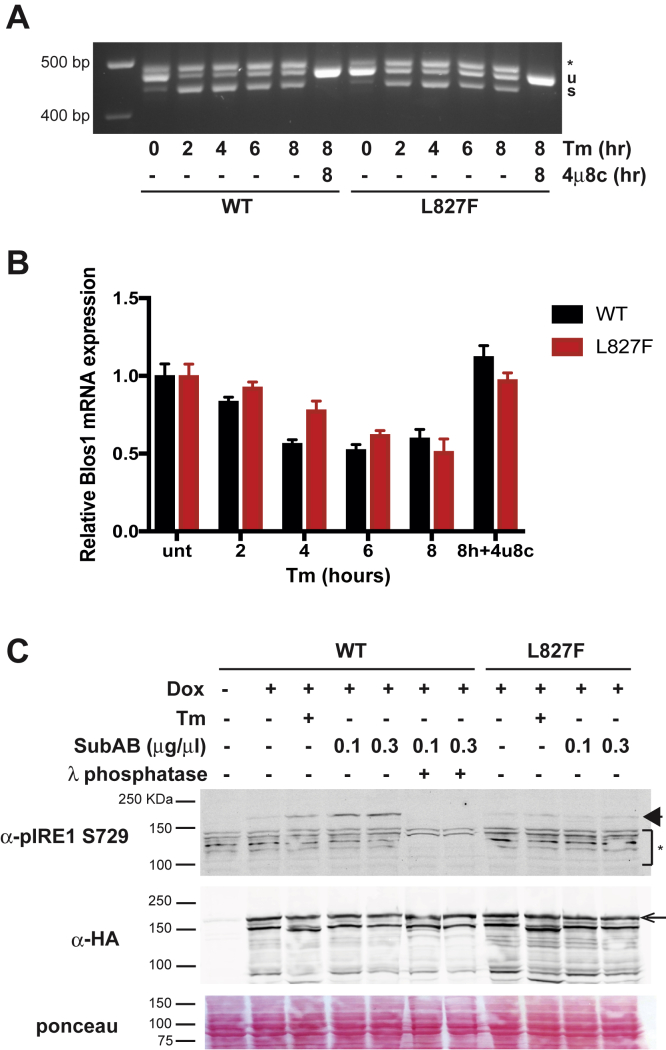
**L827F phosphorylation but not RNase activity differs from WT IRE1α**. *A*, L827F IRE1α can perform XBP1 splicing activity. HAP1KO IRE1GFP WT or L827F cells were treated with 4 μg/ml Tm or 16 μM 4μ8c for the indicated times, RNA was extracted, and XBP1 splicing was assessed by RT-PCR. Bands identity is as in Figure 1*B*. *B*, L827F IRE1α is capable of cleaving regulated IRE1-dependent decay substrates. HAP1 KO IRE1GFP WT or L827F cells were treated as in *A*, then the regulated IRE1-dependent decay activity of IRE1α was assayed using qPCR quantitation of the common BLOC1S1 substrate as in Figure 1*C*. *C*, L827F IRE1GFP is not phosphorylated on S729 following induction of endoplasmic reticulum stress. HAP1KO IRE1GFP WT or L827F was stressed with Tm (4 μg/ml) or SubAB at the indicated concentrations for 2 h. Cells were lysed, and activation of IRE1α was assessed by probing the blots first with an antibody against phosphorylated Ser729 and reprobing with anti-HA antibody. *Arrow*, full length IRE1GFP; *arrowhead*, phospho-S729 IRE1GFP; ∗, nonspecific bands.

L827F IRE1α showed interesting divergent effects on the distinct IRE1α activities; it splices XBP1 (Figs. 5*A* and 6*A*) and performs RIDD (Fig. 6*B*) as efficiently as WT IRE1α but is defective for the clustering phenotype (Fig. 5*B*). Thus, we asked how this substitution affected the activation of the kinase domain. It is surprising that the RNase activity of this mutant was induced in response to Tm without obvious phosphorylation on Ser729 (Fig. 6*C*). Therefore, we examined the general phosphorylation status of L827F IRE1α using λ phosphatase-induced electrophoretic shift, as in Figure S1*A* above. Under Tm or SubAB stress, L827F resolves into two species and the larger one is shifted by phosphatase treatment (Fig. S1*A*), indicating that it is phosphorylated, even though it is not reactive with anti-pSer729. We conclude that L827F is phosphorylated in response to ER stress but at different positions from WT IRE1α.

### Comparison of WT, L827P, and L827F IRE1α by MD simulations

Given the phenotypic differences between WT, L827P, and L827F, we sought experimental evidence for conformational changes induced by the mutations. Our first approach was computational, employing MD simulations of the structures.

To simulate conformational transitions induced in the inactive dimer by phosphorylation, we used the crystal structure of the face-to-face dimer 3p23, added phosphates in the activation loop (38), and removed the ATP and Mg^2+^ ligands to initiate unbiased 400-ns MD simulations of WT, L827P, and L827F dimers in the face-to-face conformation. Principal component analysis of the trajectories indicated variance in the coordinate system, which suggested increased flexibility in mutants IRE1α compared with WT, and it was used to visualize the conformational landscape visited by each protein.

**Figure 7:**
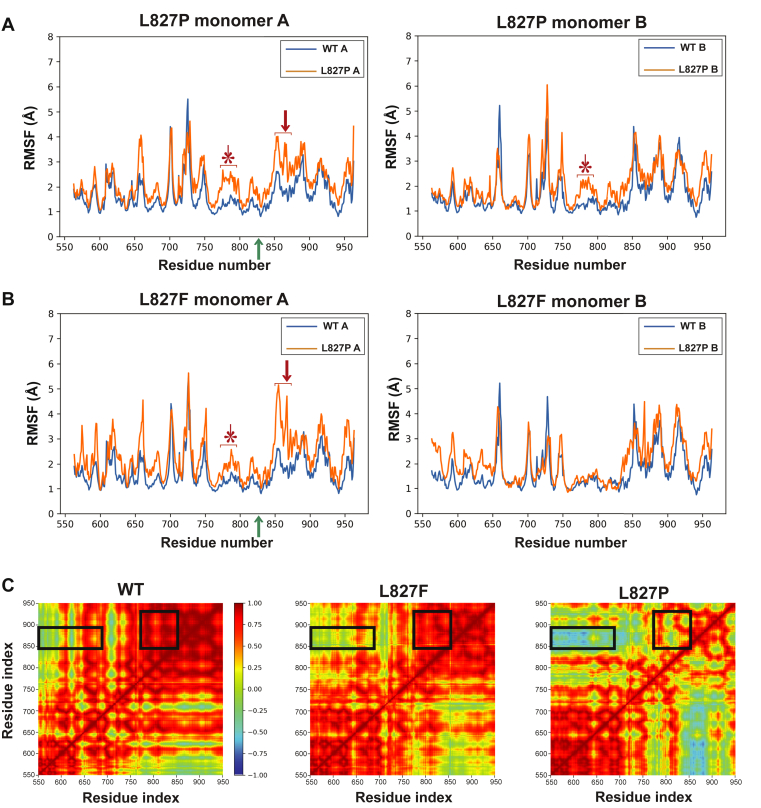
**MD simulations predict that L827P and L827F have conformations distinct from WT IRE1α**. *A*, MD predictions of relative mobility changes along IRE1α structures, caused by substituting L827P. The PDB: 3p23 structure was subjected to MD simulations, as described in Experimental procedures. Plotted is the root-mean-square fluctuation (RMSF) of amino acids as a function of sequence position. *Left*, predictions for the A monomers of PDB: 3p23; *right*, the B monomers. *Orange* tracing, mutant RMSF; *blue*, WT RMSF. *Green arrow* on the *x*-axis, the position of residue 827; *red arrow*, region of increased mobility C terminal to the mutation; ∗, region of increased mobility N terminal to the mutation. Note the overall asymmetry between the monomers. *B*, a similar MD analysis for the L827F substitution. *C*, dynamic cross-correlation map of WT and mutants IRE1α. Residue–residue-based correlated motions were calculated within a monomer of either WT, L827P, or L827F IRE1α, over the 400 ns of the MD simulation. MD, molecular dynamics.

The conformational landscapes of the L827P and L827F mutant systems differed from that of the WT IRE1α. Root-mean-square fluctuation analysis, indicating the deviation of each IRE1α residue from the mean structure, showed that both L827P and L827F had increased flexibility motion of residues 850 to 875, in the RNase domain, compared with the WT structure, as well as in residues 750 to 800 in the C-terminal lobe of the kinase domain (Fig. 7, *A* and *B*), namely, both upstream and downstream of helix αΚ. (Fig. 4*A*).

Although root-mean-square fluctuation analysis suggests increased local motion in IRE1α mutants relative to the WT protein, it does not give information about the directionality of the differences, which could explain the distinct phenotypes. To address that, we pursued cross-correlation analysis of the trajectory of the polypeptide over the time course of simulations. The resulting cross-correlation map (Fig. 7*C*) allowed the identification of the correlated and anticorrelated motions in the cytosolic IRE1α. Positively correlated residues (in red) move in the same direction during the simulation, whereas negatively correlated residues (in blue) move in the opposite direction (39).

Dynamic cross-correlation of L827P showed increased anticorrelated motion between residues 550 to 700 (in the kinase domain) and residues 850 to 900 (in the RNase domain) (Fig. 7*C*, rectangular box) and increased anticorrelated motion between residues 755 to 850 (kinase domain–RNase domain) and residues 850 to 940 (in the RNase domain) (Fig. 7*C*, square box). The dynamic cross-correlation pattern of L827F appears similar to the pattern of WT IRE1α, consistent with its quasi-normal phenotype. Thus, even though both L827P and L827F IRE1α show increased motion in the C-terminal end of the kinase domain and around amino acid 860 in the RNase domain, in the L827P mutant those residues appear to move in opposite directions, suggesting an explanation for its dramatic phenotype.

### L827P and L827F IRE1α have conformations distinct from WT IRE1α

The above calculations together with published data by Xue *et al*. (28) suggest that substitutions of amino acid 827 or 830 perturb the IRE1α structure both upstream and downstream of the mutations. Indeed, L827P and P830L affect RNase and kinase activities that are carried out by residues that are ∼70 or 230 residues removed, respectively, from the mutations. Therefore, our second approach was to seek further evidence *via* limited trypsin proteolysis. Lysates of HAP1KO IRE1GFP WT or L827P or L827F cells, either untreated or stressed with Tm, were subjected to low-dose digestions with trypsin (0.25–10 μg/ml). The resulting peptides were analyzed by Western blots with two different antibodies: anti-HA and anti-GFP (see Fig. 1*A* for location of the tags).

**Figure 8:**
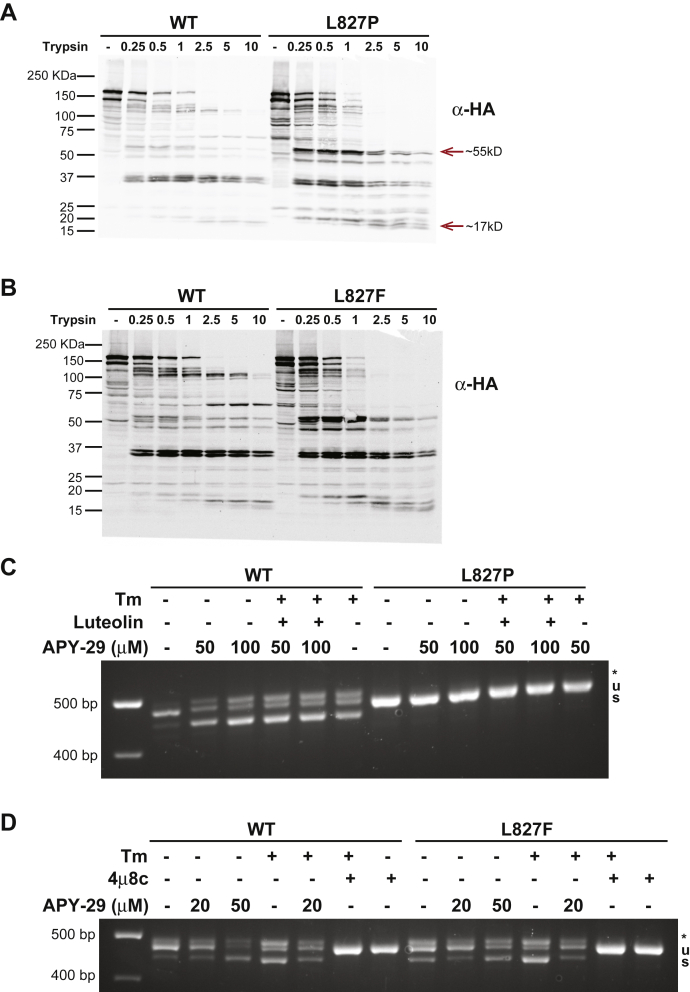
**L827P and L827F have different conformations from WT IRE1α.***A*, WT and L827P IRE1GFP have different conformations under endoplasmic reticulum stress conditions. HAP1KO IRE1GFP WT or L827P cells treated with Tm 4 μg/ml for 4 h were lysed and subjected to the indicated range of trypsin concentrations (μg/ml) for 30 min on ice. Western blot analysis was performed, and the membranes were probed with anti-HA antibody. The *red arrows* point to some of the trypsin-induced fragments that differ between WT and L827P. *B*, the L827F conformation differs from WT IRE1GFP under endoplasmic reticulum stress. The same procedure described in *A* was performed on either WT or L827F IRE1GFP. *C*, The L827P mutant is not activated by APY-29. HAP1KO IRE1GFP WT or L827P was treated with the indicated concentrations of APY-29, luteolin (50 μΜ), or Tm (4 μg/ml) for 2 h. RT-PCR to detect XBP1 splicing was performed. *D*, the L827F mutant is sensitive to APY-29. HAP1KO IRE1GFP WT or L827F was treated with the indicated concentrations of APY-29, Tm (4 μg/ml), or 4μ8c (16 μM) for 2 h. XBP1 splicing was detected as above.

Comparison of WT digest with L827P IRE1α revealed unique tryptic fragments characteristic of the L827P mutant under ER stress (Fig. 8*A*), indicating that the two molecules are differentially susceptible to trypsin treatment. For example, the ∼55-kDa HA-positive fragment, which is more prevalent in the L827P mutant, indicates a more accessible tryptic site in the N-terminal lobe of the kinase domain (Fig. 8*A*). Also, particularly informative are the ∼17-kDa peptides (Fig. 8*A*): their size indicates a trypsin site ∼154 residues upstream of the C terminus, around IRE1α residue 860, which is more accessible in the L827P mutant. The precise identification of the cleavage site awaits mass spectrometric analysis but each of the four other potential sites is well downstream of residue 827, so the conclusion will be similar. The change in accessibility of this site in the RNase domain is consistent with the changes predicted by our MD simulations and reinforce the conclusion that helix αK is a conduit for the conformational change that activates the RNase domain of IRE1α.

Other informative fragments are exemplified by the doublet at 36 kDa, which represents a site that, in unstressed cells, is more resistant to trypsin in the WT protein and more sensitive in the L827P protein (Fig. S6*A*). The size of these peptides shows that the conformation hundreds of amino acids upstream of the mutation site is altered by the L827P substitution. We conclude that distinct sites are more exposed to trypsin in L827P than in WT IRE1α under ER stress. Remarkably, when L827F was subjected to similar partial proteolysis, the proteolysis pattern resembled that of the mutant L827P and not the WT protein, despite its preserved RNase activity (Fig. 8*B* and S6*B*). One notable difference was the absence of the small C-terminal proteolytic fragments that were prominent in the L827P digests, suggesting that the distant conformational changes in these two mutants are not the same. Thus, the trypsin sensitivity assay mirrors the phosphorylation and clustering activities more than the RNase activity.

To independently confirm that mutations in helix αK lead to different conformations at distant sites, we utilized the allosteric inhibitor of the kinase domain, APY-29 (20, 40), that provides a paradoxical bypass activation mode for IRE1α, by conferring an allosteric conformational change in the kinase domain. Paradoxically, APY-29 activates the XBP1 splicing activity in cells at concentrations above 20 μM, even without the stressor Tm (Fig. 8, *C* and *D*), converting IRE1α to an RNase-active, kinase-inactive conformation (20) as well as to a higher oligomeric state. The L827P mutant was refractive to APY-29 treatment (Fig. 8*C*), whereas L827F responded to APY-29 (Fig. 8*D*). The different behaviors suggest that despite its conformational similarity to L827F (Fig. 8, *A* and *B*), L827P locks IRE1α in a nonresponsive conformation.

The trypsin sensitivity assay and the response to the allosteric inhibitor confirm that the interdomain connector can impart distinct conformations on IRE1α, some of which can be manipulated using allosteric kinase inhibitors.

## Discussion

The mutational analysis in this article defines an intramolecular relay through which the kinase domain of IRE1α communicates to the RNase domain to activate the stress sensor. Following the trans-autophosphorylation that occurs when IRE1α dimerizes, a conformational change is relayed through residues L827–W833 in helix αK (data here and in (28)). Mutations in helix αK affect all the measurable activities of IRE1α: XBP1 splicing, RIDD activity, homo- and hetero-oligomerization, and thereby impact the quality of IRE1α response to ER stress. Even though helix αK is in the cytosolic portion of IRE1α and not in the luminal domain or in the transmembrane segment, which are each known to sense ER stress, it is necessary for proper activation of IRE1α.

We show that mutations in helix αK cause IRE1α to assume distinct conformations in response to ER stress, different from the conformations of WT IRE1α. The distinct conformations of IRE1α WT and mutants in residue 827 were predicted by MD simulations of the proteins and were confirmed experimentally. The most detrimental substitution is L827P, which abolishes the autophosphorylation of the sensor and its ability to splice XBP1, to cleave RIDD transcripts, and to cluster in response to ER stress. L827P is also refractive to the bypass activation of IRE1α by flavonoids (16) and by allosteric kinase inhibitors (20, 36). L827 is adjacent to the previously described loss-of-function mutations P830L and W833A (28). We show that these three amino acids and their interacting residues form an evolutionarily conserved, functionally important signaling relay element between the kinase and RNase domains. Of importance, even the most severe mutant, L827P, is not a misfolded protein, since it is still able to dimerize with WT IRE1α and inactivate it (*e.g.*, Fig. 2).

Other substitutions of L827 and P830 (L827R, L827F, L827Q, and P830A) yield intermediate phenotypes, consistent with the predicted effects of these substitutions on the structure of IRE1α. Furthermore, the less severe substitutions distinguish the inhibition of clustering from the inhibition of RNase activity and reinforce previous data showing that ER stress-responsive clustering of IRE1α is a distinct manifestation of activation (23).

The phenotypes of all these substitutions suggest an effect due to altered backbone conformation of this connecting segment. Based on the crystal structures, L827P is predicted to (a) alter the backbone conformation of helix αΚ between the kinase domain and the RNase domain (Fig. 4); (b) create steric clashes with residues A823, H825, and V826 at the C-terminal end of the kinase domain; and (c) disrupt the proximity of L827 to residues T673 and A677 in helix αE of the kinase domain. It stands to reason that such local effects of the mutations in interdomain helix αΚ would alter the positioning of the RNase domain relative to the kinase domain, perhaps determining the face-to-face or back-to-back orientation of the IRE1α monomers and therefore affecting the activation of the RNase domain (18, 19). However, an important caveat is that the crystal structures of WT human IRE1α in the apo form (5HGI (36)), the nonphosphorylated form (3P23 (17)), and phosphorylated pIRE1α (4YCZ and 4YZ9 (41)), all show the helix αK at essentially super-imposable conformations. Thus, the conformational change that our experimental data and MD simulations imply has not yet been captured in the X ray structures of IRE1α.

The transmission of conformational changes along the axis of the IRE1α molecule had long been considered to require autophosphorylation at several sites (38), but it was later shown that the phosphoryl transfer *per se* is not essential (18, 19, 20). Instead, the activated kinase initiates a conformational change that is transmitted to the RNase domain. The phosphorylation requirement can be bypassed by mutations like I642G (18) or by using effector small molecules like APY-29 (20), both changing the kinase active site itself. The kinase-to-RNase conformational change is not well defined at present, and our work suggests that it involves helix αK. None of the known phosphorylation sites (38) are in or near this helix and yet L827P and P830L are not phosphorylated and L827F is phosphorylated in a noncanonical manner. Therefore, it seems that the interdomain helix αΚ can adopt several intermediate conformations needed to activate the RNase domain, only some of which are captured in the current crystal structures. Since some of these conformations are compatible with enzymatic activity but incompatible with oligomerization of IRE1α, the kinase-RNase domain interface may behave as a tuner that can allow distinct outcomes of activating the IRE1α sensor.

Since crystallography may miss some intermediate conformations needed to activate the RNase domain, we followed the motion of IRE1α WT and L827P or L827F in MD simulations (Fig. 7). We found increased motion in areas far from the vicinity of the mutation: around aa 860 in the RNase domain and in the C-terminal lobe of the kinase domain. This also suggests that some regions are more accessible to trypsin digestion in the mutants (Fig. 8). Of note, the directionality of the motion differed between the two mutants. These residues appear to move in opposite directions only in the L827P mutant, possibly explaining why this mutation is the most detrimental one.

There are subtle but potentially important differences among loss-of-function mutants in helix αΚ. First, unlike L827P, P830L and W833A were not shown to physically interact with WT IRE1α; this likely explains why neither of them acted in a dominant-negative fashion when IRE1α activation was assayed by phosphorylation or oligomerization (20). Second, P830L and W833A IRE1α were degraded more rapidly than WT (28), whereas the turnover of L827P mutant is unchanged. Our analysis of the predicted free energies of the various substitutions (Table 1) suggests that P830L is a less severe mutation than L827P, so perhaps the phenotypic differences reflect distinct cell expression environments used in this work and in Xue *et al*. (28) and/or differential affinity of P830L monomers to WT monomers. A third interesting difference between P830L and L827P is their responsiveness to the kinase inhibitor APY-29: even though both of these mutants are RNase inactive, the phosphorylation activity of P830L is *decreased* by APY-29 and its oligomerization is *increased* (20), whereas L827P is refractive to this drug treatment. The differential responsiveness of these IRE1α mutants suggests that their conformations are distinct.

The dominant-negative nature of L827P is evident not only when overexpressed in fibroblasts but also in relevant lymphoid cell lines where the mutants are expressed at nearly normal level. The self-association of IRE1α monomers is known to be mediated by the luminal domains as well as by the transmembrane segments and the cytoplasmic domains (11, 15). The inability of the WT endogenous protein to phosphorylate the mutant protein suggests that the kinase domains in the mixed dimers are not oriented properly for transphosphorylation of the mutant by WT. This dominant-negative phenotype leads to hypersensitivity to chemical ER stress, underscoring the importance of the IRE1α stress sensing pathway for survival of leukemias and lymphomas (42, 43, 44, 45). So far, no dominant-negative IRE1α has been described in patients or animals, but the mutants described here suggest that there are binding sites outside the kinase or RNase active sites that can be targeted to mimic the proapoptotic conformation seen in the L827P mutant. Moreover, the IRE1α kinase active site is structurally similar to other kinases, and therefore targeting a connecting segment whose function depends on kinase activity might be a new selective strategy.

## Data availability

All the data are contained within the article.

## Supporting information

This article contains supporting information (23).

## Conflict of interest

The authors declare that they have no conflicts of interest with the contents of this article.
