# Supplementary material for: An interdomain helix in IRE1α mediates the conformational change required for the sensor's activation
Source: J Biol Chem. 2021 May 14;296:100781. doi: 10.1016/j.jbc.2021.100781 (PMC8203841; doi:10.1016/j.jbc.2021.100781)
Supplement: Figures S1 to S6 [file mmc1.pdf]

Suppl. Fig. 1

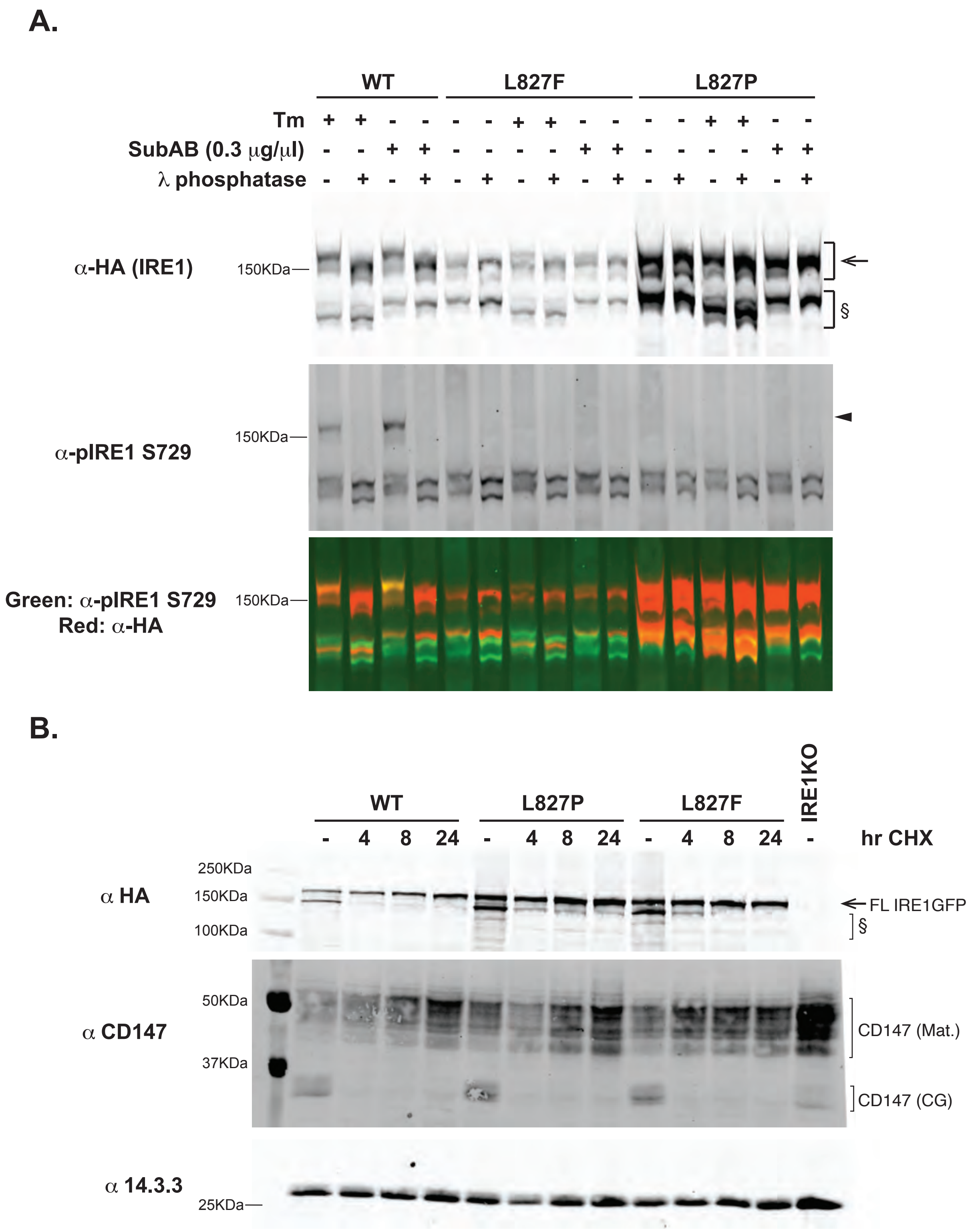
A.
WT
L827F
L827P
Tm
SubAB (0.3 μg/μl)
λ phosphatase
α-HA (IRE1)
150KDa
§
α-pIRE1 S729
150KDa
Green: α-pIRE1 S729
Red: α-HA
150KDa
B.
WT
L827P
L827F
IRE1KO
hr CHX
- 4 8 24 - 4 8 24 - 4 8 24 -
α HA
250KDa
150KDa
100KDa
FL IRE1GFP
§
α CD147
50KDa
37KDa
CD147 (Mat.)
CD147 (CG)
α 14.3.3
25KDa

Suppl. Fig. 2

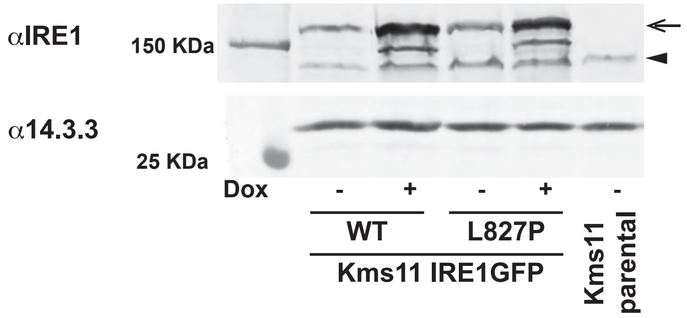

αIRE1
150 KDa
α14.3.3
25 KDa
Dox
-
+
-
+
-
WT
L827P
Kms11 IRE1GFP
Kms11
parental

Suppl. Fig. 3

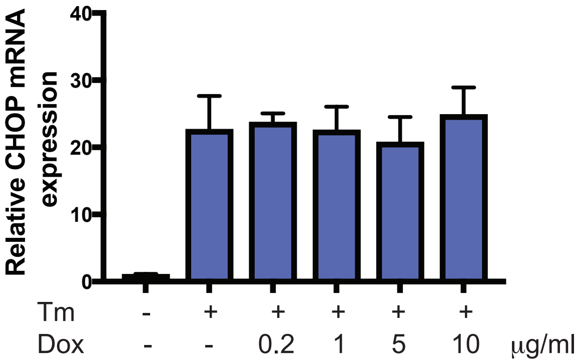
Relative CHOP mRNA expression
40
30
20
10
0
Tm - + + + + +
Dox - - 0.2 1 5 10 μg/ml

Suppl. Fig. 4

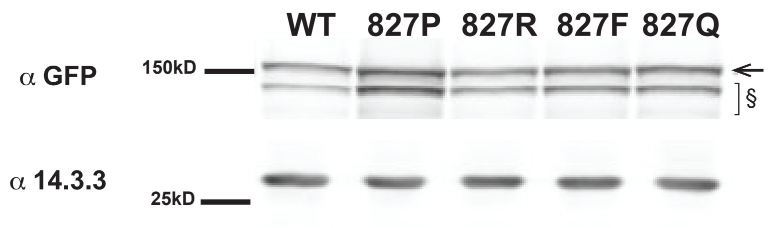

Suppl. Fig. 5

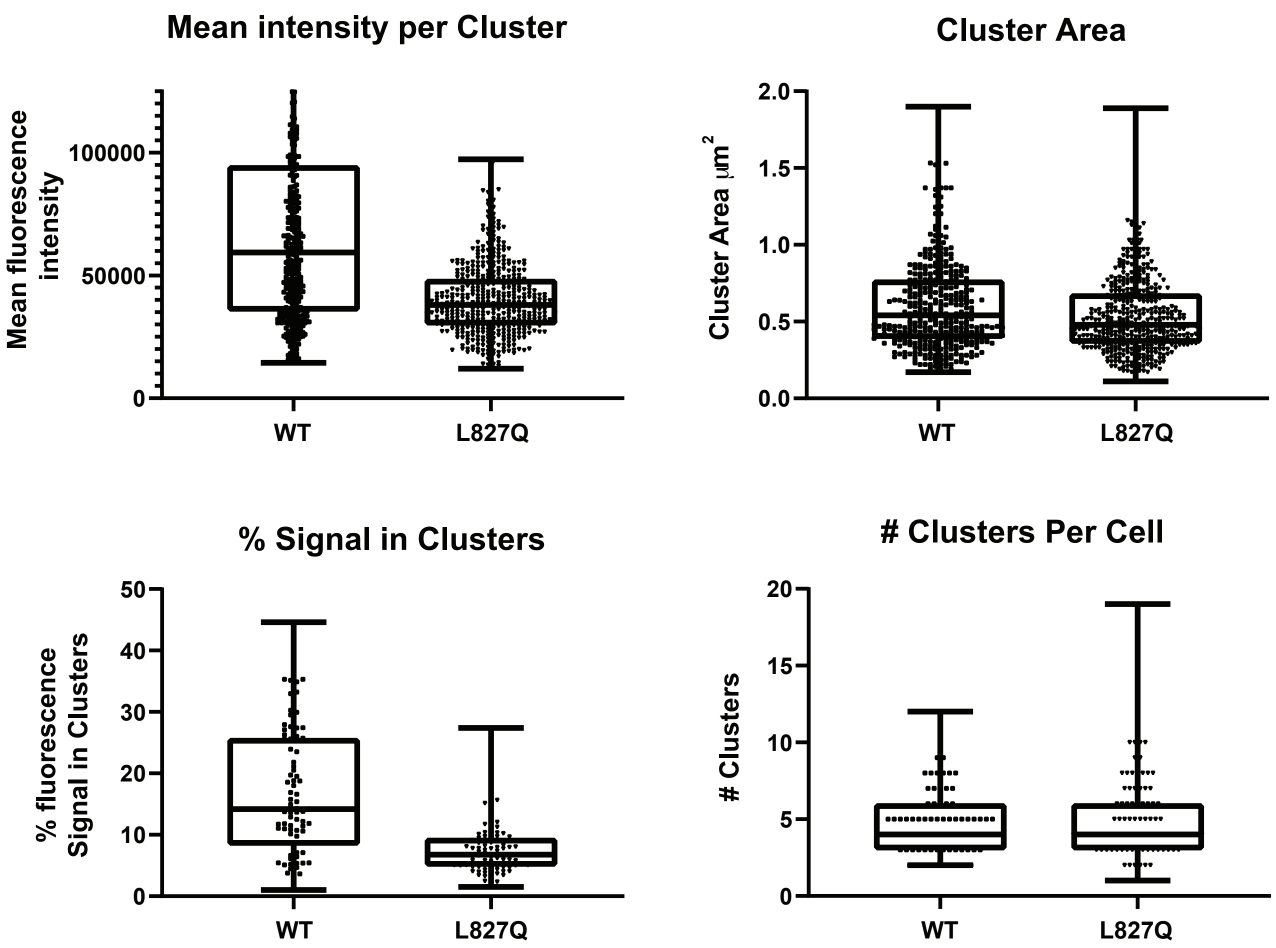

Mean intensity per Cluster
Mean fluorescence intensity
100000
50000
0
WT
L827Q
Cluster Area
Cluster Area μm2
2.0
1.5
1.0
0.5
0.0
WT
L827Q
% Signal in Clusters
% fluorescence Signal in Clusters
50
40
30
20
10
0
WT
L827Q
Clusters Per Cell
Clusters
20
15
10
5
0
WT
L827Q

Suppl.Fig. 6

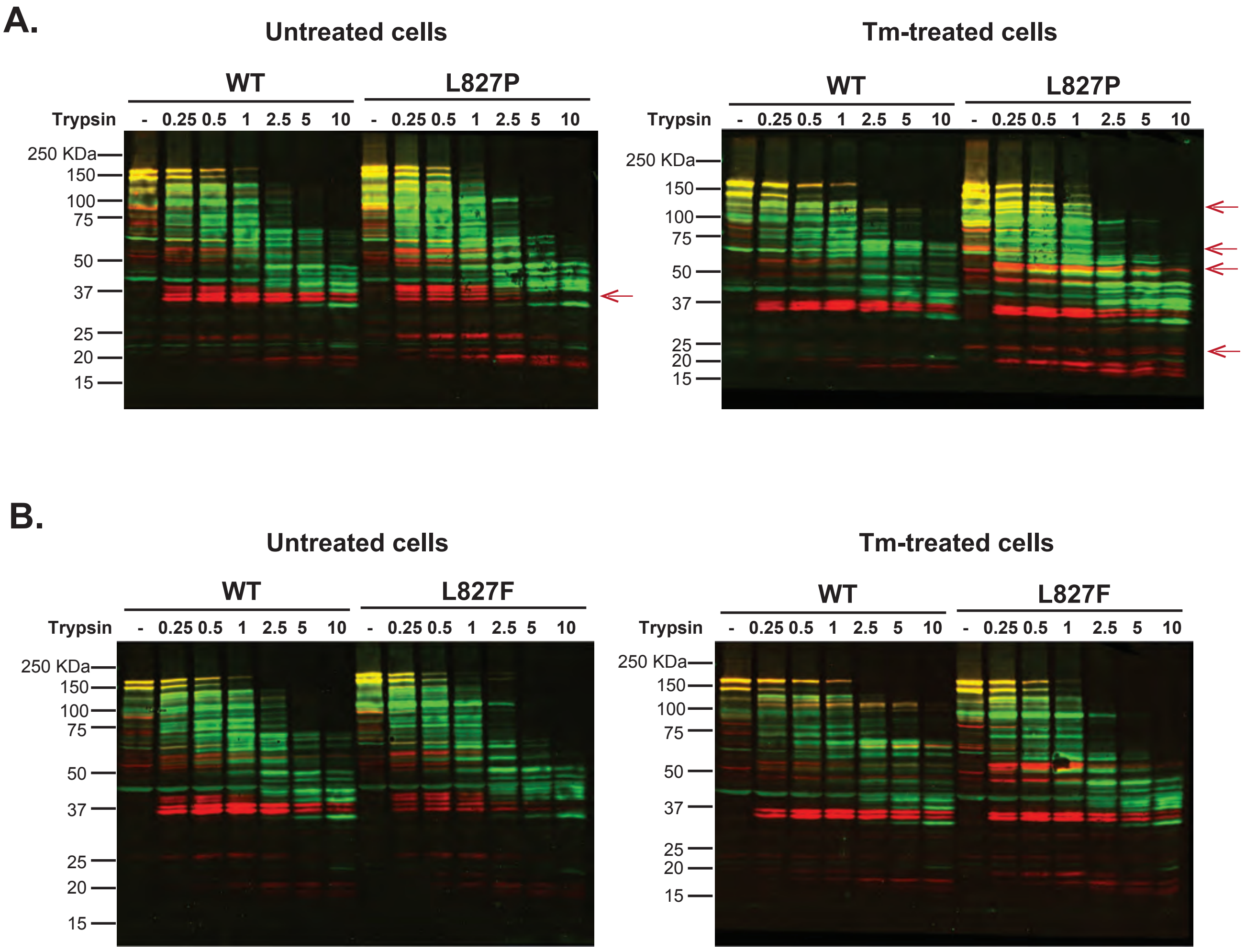
A.
Untreated cells
Tm-treated cells
WT
L827P
Trypsin
- 0.25 0.5 1 2.5 5 10
250 KDa
150
100
75
50
37
25
20
15
B.
Untreated cells
Tm-treated cells
WT
L827F
Trypsin
- 0.25 0.5 1 2.5 5 10
250 KDa
150
100
75
50
37
25
20
15

Supplementary Figure Legends

Suppl. Figure 1. **Phosphorylation levels and protein stability of L827P, L827F IRE1α mutants.**

A. HAP1KO IRE1GFP WT, L827F or L827P were treated with Tm (4 μg/ml) or SubAB (0.3 μg/ml) for 2 hr. The cells were lysed, subjected to phosphatase treatment for 30 minutes at 30°C where indicated and proteins were analyzed by western blot. Arrow: full-length IRE1GFP; §: lower molecular weight species; arrowhead: phosphor-IRE1GFP S729.

B. Cycloheximide chases (10 μg/ml over 24 hr) of HAP1KO cells expressing WT IRE1GFP, L827P or L827F. Cells were lysed and proteins analyzed by western blot. Arrow indicates full length IRE1GFP; §: lower molecular species which appear to be cycloheximide sensitive. Anti-CD147 antibody was used as a positive control for cycloheximide treatment. Mat.: mature form of CD147; CG: core glycosylated CD147. 14.3.3: housekeeping protein.

Suppl. Figure 2. **Expression levels of exogenous IRE1GFP in multiple myeloma Kms11.**

Expression levels of WT and L827P IRE1GFP proteins in Kms11 cells. Kms11 parental cells or expressing WT or L827P IRE1GFP were induced with dox for 16 hr. Cells were lysed and protein were subjected to western blot analysis. Arrow: full-length IRE1GFP; arrowhead: endogenous IRE1α.

Suppl. Figure 3. **IRE1α L827P does not affect endogenous PERK activity.**

HAP1 cells expressing endogenous IRE1α were complemented with L827P IRE1GFP and exposed to different amounts of dox for 16 hr where indicated. Cells were then treated with Tm (4 μg/ml) for 4 hr, RNA was extracted and relative CHOP mRNA expression was assayed using RT-qPCR quantitation. Data are expressed as the relative abundance of CHOP under each condition relative to the abundance of the unaffected ribosomal gene Rpl19.

Suppl. Fig. 4. **Expression levels of L827 IRE1 mutants.**

Expression of L827 mutants. HAP1KO cells were infected with lentiviruses expressing the indicated IRE1GFP constructs and selected by puromycin to obtain stable clones. Detergent lysates of each were resolved by SDS-PAGE and probed with anti-GFP. Anti-

14.3.3 probing served as a loading control. Arrow: full length IRE1GFP; §: lower molecular weight bands that appear to be IRE1α specific.

Suppl. Figure 5. <u>L827Q have mild differences with WT IRE1α for ER-stress induced clustering</u>.

The ER-stress induced L827Q clusters show some differences with WT ones. HAP1KO IRE1GFP WT or L827Q cells were induces with dox for 16 hr and treated with Tm (4μg/ml) for 4 hr. Images were taken and analyzed using a home-made cluster analyzer for ImageJ (Ricci et al., 2019). Several parameters were taken into consideration and plotted.

Suppl. Figure 6. <u>L827P and L827F IRE1α have distinct conformations in both unstressed and stressed conditions.</u>

A. WT and L827P IRE1GFP have different conformation. HAP1KO IRE1GFP WT or L827P cells untreated (left panel) or treated with Tm 4 μg/ml for 4 hours (right panel) were lysed and subjected to the indicated range of trypsin concentrations (μg/ml) for 30 min on ice. Western blot analysis was performed and the membranes probed with anti-GFP (in green) and anti-HA (in red). Yellow bands contain both tags. The GFP domain is 27.5kD. The red arrows point to trypsin-induced fragments that differ between WT and L827P.

B. L827F conformation differs from WT IRE1GFP. The same procedure described in A was performed on either WT or L827F IRE1GFP.
